# Assessment of cytology based molecular analysis to guide targeted therapy in advanced non-small-cell lung cancer

**DOI:** 10.18632/oncotarget.6671

**Published:** 2015-12-18

**Authors:** Wenbin Li, Zhihui Zhang, Lei Guo, Tian Qiu, Yun Ling, Jian Cao, Huiqin Guo, Huan Zhao, Lin Li, Jianming Ying

**Affiliations:** ^1^ Department of Pathology, Cancer Hospital, Chinese Academy of Medical Sciences and Peking Union Medical College, National Cancer Center, Beijing, China

**Keywords:** ALK, fluorescence in situ hybridization, EGFR, targeted therapy, patient outcomes

## Abstract

To investigate the use of molecular testing on cytological specimens in selecting advanced non-small cell lung cancer (NSCLC) patients who are adequate for targeted treatment, a total of 137 NSCLC cases were analyzed by fluorescence *in situ* hybridization (FISH) for anaplastic lymphoma kinase (*ALK*) rearrangements, and Epidermal growth factor receptor (*EGFR*), kirsten rat sarcoma viral oncogene homolog (*KRAS*) mutations were evaluated by quantitative real-time PCR (qRT-PCR) platform combining amplification refractory mutation system (ARMS) primers and TaqMan probes. Cytological specimens included 91 fine-needle aspirates, 5 fibreoptic bronchoscopic derived samples and 41 pleural effusions. Among 137 NSCLCs analyzed for *ALK* FISH, 16 (11.7%, of 137) were detected to harbor *ALK* rearrangement. FISH positive cases were all defined as adenocarcinoma (ADC) histologic subtype and the FNA samples showed the highest *ALK* positive rate (13.2%, 12/91). Of the 9 *ALK* FISH positive patients who received crizotinib treatment, 8 (88.9%) patients exhibited tumor regression. In addition, 60 (44.8%, of 134) cases were found to harbor *EGFR* mutations and 22 patients with *EGFR* sensitive mutations who received gefitinib or erlotinib treatment showed a median PFS of 16.0 months. Mutations of *KRAS* occurred in 8 (6.0%, of 134) cases and this was mutually exclusive from *EGFR* mutation. Our results demonstrated that *ALK* FISH and *EGFR, KRAS* mutational analysis on cytological specimens are sensitive methods for screening advanced stage NSCLC patients who are adequate for targeted treatment.

## INTRODUCTION

Non-small-cell lung cancer (NSCLC), which accounts for approximately 85% of lung cancers, has been largely identified by oncogenic driver mutations with potential opportunities for targeted therapies [1, 2]. Recently, activation of the anaplastic lymphoma kinase (*ALK)* gene in lung cancer by fusion to echinoderm microtubule-associated protein-like 4 (*EML4*) or other gene partners (such as *BIRC6* [3]*, TFG* [4]*, KIF5B* [5] and *KLC1* [6]) has been identified as oncogenic events [7]. Clinical studies have shown that locally advanced or metastatic NSCLC patients harboring *ALK* gene rearrangement are highly sensitive to Crizotinib, which is a small molecular inhibitor of *ALK* tyrosine kinase [8, 9]. In addition, NSCLC with sensitive epidermal growth factor receptor (*EGFR*) mutations are well responded to tyrosine kinase inhibitors (TKI) and these patients will have a longer progression-free survival (PFS) than the patients whose tumors do not contain *EGFR* mutations [1, 10].

Approximately 60% of patients with NSCLC are diagnosed at a late stage for the first time [11]. These patients are not suitable for the resection of the primary tumor, and the only pathologic material guiding systemic therapy should be small biopsy or cytological specimens. Recent studies have demonstrated that cytological specimens, including fine-needle aspiration (FNA), fibreoptic bronchoscopic (FOB) and pleural effusion (PLE), are suitable for the molecular testing [12–16]. Although FISH (fluorescent *in situ* hybridization) is currently the gold standard method to detect *ALK* gene rearrangement approved by FDA, its application on cytological specimens remains a worth area of investigation. In this study, we investigate the use of *ALK* FISH and *EGFR*, *KRAS* mutational testing on cytological specimens and to evaluate PFS of the patients who received targeted therapies.

## RESULTS

### Specimen and patient characteristics

Demographic and clinicopathologic features were summarized in Table 1. Of the 137 patients enrolled in the study, 54 (39.4%, of 137) were male and 83 (60.6%, of 137) were female. The mean age at diagnosis was 58.8 years (range: 27.0 - 85.0 years) and the median age was 59.0 years. Cytological specimens (*n* = 137) included FNAs (*n* = 91), FOBs (*n* = 5) and PLEs (*n* = 41). Of these, 126 (92.0%, of 137) were diagnosed as ADC, 3 (2.2%, of 137) as SCC, 1 (0.7%) as adenosquamous carcinoma and 7 (5.1%, of 137) as NSCLC, not otherwise specified.

**Table 1:** Demographic and clinicopathologic features of the study patients

Characteristics	*No.*	Percent
Sex		
Male	54	39.4%
Female	83	60.6%
Sample type		
FNA	91	66.4%
FOB	5	3.6%
PLE	41	30%
Histologic type		
ADC	126	92.0%
SCC	3	2.2%
Adenosquamous	1	0.7%
NSCLC, NOS	7	5.1%
Age		
Mean (SD)	58.8 ± 12.1	
Median	59.0	
Range	27.0–85.0	
*ALK* FISH		
Positive	16	11.7%
Negative	121	88.3%
*EGFR* mutation status		
Sensitive mutation	53	39.5%
Exon 20 mutation	4	3.0%
Complex mutation with exon 19 deletion + T790M	1	0.7%
Complex mutation with L858R + T790M	2	1.5%
Negative	74	55.3%
Not tested	3	NA
*KRAS* mutation status		
Positive	8	6.0%
Negative	126	94.0%
Not tested	3	NA

Abbreviations: *ALK* = anaplastic lymphoma kinase; FISH = fluorescence *in situ* hybridization; FNA = fine needle aspiration; FOB = fibreoptic bronchoscopic; PLE = pleural effusion; ADC = adenocarcinoma; SCC = squamous cell carcinoma; NSCLC, NOS = non-small cell lung cancer, not otherwise specified.

### *ALK* FISH analysis

**Figure 1:**
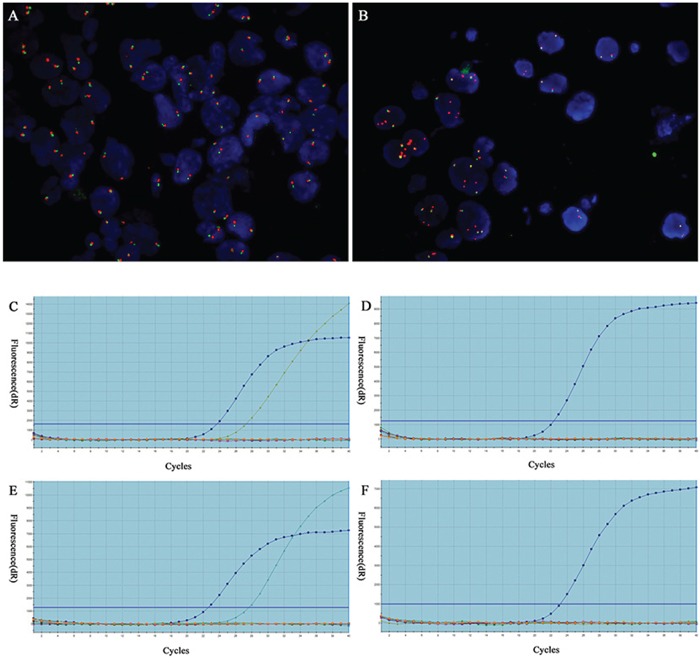
Detection of *ALK* fusion by FISH and *EGFR/KRAS* mutations in cytological specimens by qRT-PCR **A.** Representative image of FISH negative case showing intact two fused signals per nucleus. **B.** Representative image of FISH carried out with Vysis LSI *ALK* Dual color Break-Apart FISH probes detected *ALK* fusion as split red and green signals. Original magnification ×1000. **C-D.** Detection of *EGFR* and *EGFR* L858R mutation and negative case. **E-F.** *KRAS* p.G12D mutation and negative case.

Of the 137 NSCLCs analyzed for *ALK* FISH, 16 (11.7%, of 137) were detected to harbor *ALK* rearrangement (FISH positive) and 121 (88.3%, of 137) were FISH negative. The *ALK* FISH positive cases of the cytological samples were 12 (75.0%, of 16) FNAs, 1 (6.2%, of 16) FOB and 3 (18.8%, of 16) PLE (Supplementary Table S1). The FNA samples showed highest FISH positive rate (13.2%, 12/91) among three groups, although this did not demonstrate a statistically significant difference (*P* = 0.32). On FISH examination (Figure 1), split pattern was observed in 14 cases (87.5%) and unbalanced rearrangement, characterized by a loss of the 5′ probe, was shown in 2 cases (12.5%).

The *ALK* FISH positive cases included 6 men and 10 women and there was no significant difference in gender distribution between FISH positive and negative cases (*P* = 0.87). The mean age at diagnosis for FISH positive cases was 52.7 ± 11.8 years, which was much younger than that of FISH negative cases (*P* < 0.05). FISH positive cases were all defined as ADC histologic subtype. In addition, there was one FISH positive case also demonstrated an *EGFR* L858R mutation (Table 2).

**Table 2:** Clinicopathologic characteristics of *ALK* FISH positive and negative cytology cases

Characteristics	*ALK* FISH positive (n = 16)	*ALK* FISH negative (n = 121)	*P* value
Sex			0.87†
Male	6 (37.5%)	48 (39.7%)	
Female	10 (62.5%)	73 (60.3%)	
Histologic type			NA
ADC	16 (100%)	110 (90.9%)	
SCC	0	3 (2.5%)	
Adenosquamous	0	1 (0.8%)	
NSCLC, NOS	0	7 (5.8%)	
Age			0.03‡
Mean (SD)	16	59.6 ± 11.9	
Median	53.5	60.0	
Range	27.0 – 73.0	30.0 – 85.0	
*EGFR*			0.002†
Positive	1 (6.7%)	59 (49.6%)	
Negative	14 (93.3%)	60 (50.4%)	
*KRAS*			0.42§
Positive	0	8 (6.7%)	
Negative	15 (100%)	111 (93.3%)	

Abbreviations: *ALK* = anaplastic lymphoma kinase; FISH = fluorescence *in situ* hybridization; ADC = adenocarcinoma; SCC = squamous cell carcinoma; NSCLC, NOS = non-small cell lung cancer, not otherwise specified.

† Two-sided χ^2^ test

‡ Two-sided Kruskal Wallis test

§ Fischer's exact test

### *EGFR* and *KRAS* mutation status

Among 134 NSCLCs tested, 60 (44.8%, of 134) cases carried *EGFR* mutations, which was 53 (39.5%, of 134) sensitive mutations and 4 (3.0%, of 134) exon 20 mutations with S768I. There was one case demonstrated complex mutation with exon 19 deletion and T790M and the other two cases showed complex mutation with L858R and T790M. Among them, two patients carried acquired T790M mutation after TKI treatment and one patient carried primary coexisting mutations of T790M and L858R. In addition, four patients carried primary exon 20 S768I mutations and did not receive targeted therapies. The mutated *EGFR* cases of the cytological samples were 36 (60.0%, of 60) FNAs, 1 (1.7%, of 60) FOB and 23 (38.3%, of 60) PLEs (Supplementary Table S2). The PLE samples showed highest *EGFR* mutation rate (58.9%, 23/39) among three groups, although this did not demonstrate a statistically significant difference (*P* = 0.41). Mutations of *KRAS* occurred in 8 (6.0%, of 134) cases and this was mutually exclusive from *EGFR* mutation. In addition, compared with male patients, female patients were more likely to carry *EGFR* mutations (55.6% *vs* 28.3%, *P* = 0.002) (Supplementary Table S3).

### Outcomes of targeted therapies

Of the 9 patients who received crizotinib treatment, there were 5 men and 4 women with mean age of 58.6 years (Table 3). The median follow-up duration was 9 months (range: 6–15 months). At the end of follow up, 8 (88.9%) patients were still receiving crizotinib treatment and there was one person stopping the treatment because of disease progression.

**Table 3:** Progression-free survival of crizotinib treatment *ALK* FISH positive patients

Case no.	Age	Sex	Type of samples	PFS (months)	Status
1	55	Female	PLE	8	PR
2	48	Male	FNA	5	PD
3	72	Male	FNA	12	PR
4	42	Female	FNA	3	PR
5	46	Female	FNA	5	PR
6	51	Male	FNA	6	PR
7	27	Male	FNA	5	PR
8	62	Female	PLE	4	PR
9	55	Male	PLE	4	PR

Abbreviations: *ALK* = anaplastic lymphoma kinase; FISH = fluorescence *in situ* hybridization; FNA = fine needle aspiration; PLE = pleural effusion; PFS = progression-free survival; PR = partial response; PD = progressive disease.

We performed targeted next-generation sequencing (NGS) in one case who did not response well to crizotinib treatment. Results confirmed the existence of *EML4*-*ALK* transloction, and the relative abundance of *EML4-ALK* fusion was 25.3%. In addition, there were a nonsense mutation (c.991 C > T, p.Q331) in exon 9 of *P53* and a deletion of *CDKN2A*. The mutation in *P*53 could result in a stop codon and lead to loss of *P53* function as a transcription factor. [17]

**Figure 2:**
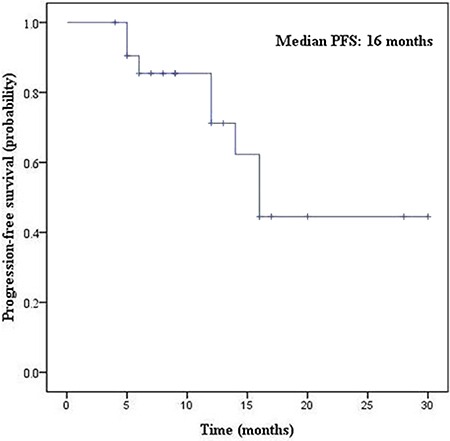
Kaplan-Meier survival curves of PFS for EGFR-TKI treatment

There were 22 patients with *EGFR* sensitive mutations who received gefitinib or erlotinib treatment. The median PFS was 16.0 months (95% confidence interval, 12.9 - 19.1) (Figure 2). Of them, 8 patients (36.4%) exhibited disease progression after 3 months due to acquired resistance to EGFR-TKI treatment. At the data cutoff point, 14 patients were still taking TKI treatment and tumor regression was observed in these patients.

## DISCUSSION

The diagnosis of advanced-stage NSCLC is usually based on a small amount of cytological specimens, consequently, the use of *ALK* FISH and *EGFR*, *KRAS* mutational testing on cytological specimens is gradually becoming a necessity in the routine molecular pathological diagnosis. Although FISH is considered to be the gold standard for detection of *ALK* rearrangements on histological tissues approved by FDA, its application on cytological specimens remains a worth area of investigation. In this study, for the first time, we demonstrated the possibility of using *ALK* FISH and *EGFR*, *KRAS* mutational testing on cytological specimens in a large cohort of Chinese patients and correlate the analysis results with PFS in patients with targeted therapies.

Targeted therapies are mostly effective in patients with locally advanced or metastatic disease, thus, molecular analysis on cytological samples rather than on resected specimens would be preferred on patients with late stage tumors. The data in previous studies demonstrated that the performance of cytology based *EGFR* and *KRAS* mutational analysis was at least as effective as histology based analysis [18–20]. In this study, various cytological specimens including FNA, FOB and PLE were all submitted for *EGFR/KRAS* mutational analysis and 97.8% (134/137) of samples were suitable for test. The other three samples not suitable for analysis were due to inadequate cell numbers and this prompted us to use more sensitive methods, such as digital PCR and NGS, to test *EGFR* mutations. Recently, a large cohort of mutational analysis on histological specimens was performed in 5125 Chinese patients and revealed that 36.2% and 8.4% patients with NSCLC carried *EGFR* and *KRAS* mutations respectively [21]. Cytological specimens of our study demonstrated slightly higher *EGFR* mutational rates (44.8% *vs* 36.2%) than that of histological specimens in above study and this could partly be attributed to the patients undertaken cytological mutational analysis often underwent a late stage disease.

Previous studies had rarely evaluated the EGFR-TKI treatment responses on patients with sensitive *EGFR* mutations tested on cytological specimens, although the outcome of the patients treated with TKI was important to confirm the predictive value of cytological mutational analysis. In our study, fifty-seven patients with *EGFR* mutations demonstrated an ADC subtype and this population would largely benefit from targeted therapies. Twenty-two patients with ADC subtype and *EGFR* sensitive mutations received TKI treatment with a median PFS of 16 months and all the patients were observed with tumor regression after 3-months treatment. This was in accordance with previous studies, which demonstrated that the median PFS interval of 16.2 months was observed in 11 *EGFR* mutated patients who treated with gefitinib [22]. In addition, Maria D. Lozano also observed patients with *EGFR* mutations had a 12.3-months median PFS [23]. These results indicated that *EGFR* mutational testing on cytological samples was adequate for patient selection with EGFR-TKI treatment. In addition to guide the targeted therapy for patients with unresected tumors, another possible advantage of cytological mutational analysis was to identify genetic shifts of *EGFR* acquired mutations during TKI treatment.

It is currently accepted that FISH is the most appropriate method to detect *ALK* rearrangements and to guide crizotinib treatment. Only a few reports have assessed the adequacy of cytological specimens for *ALK* FISH analysis, thus, its application on cytological specimens still needs further studies. Weiya Wang, et al analyzed a cohort of 58 patients for *ALK* rearrangements using pleural effusion cell blocks and found 10.3% positive cases by *ALK* FISH [16]. Agnese Proietti, et al demonstrated 4.4% cases with *ALK* FISH rearrangements in both cell blocks and small biopsies [24]. In addition, MJ. Neat, et al compared FISH and IHC methods to screen for *ALK* status in endobronchial ultrasound (EBUS)-transnronchial needle aspiration (TBNA) derived cytological specimens and found that FISH was superior to IHC for the detection of ALK rearrangement in these cytological samples [25]. To best of our knowledge, this is the largest report of *ALK* detection in cytological specimens in Chinese patients and first described the outcome of patients with *ALK* FISH positive who received crizotinib treatment. In this study, we detected 11.7% *ALK* rearrangement in 137 cases of various cytological specimens, including FNA, FOB and PLE, which was significantly higher than the rate of 6.8% previously reported in a large Chinese consecutive case series [26]. Our results were in accordance with previous report that 12.7% of ALK rearrangements were observed in malignant pleural effusion cell blocks from patients with advanced NSCLC [16]. The increased percentage of positive cases was likely attributed to the advanced stage of disease among patients with cytological specimens. Of the nine patients who received crizotinib treatment, one patient exhibited disease progression after 5 months and eight patients were still receiving crizotinib treatment at the end of cutoff point. We performed targeted next-generation sequencing in one case who did not response well to Crizotinib treatment and found that there was a nonsense mutation in exon 9 of *P53* which could lead to loss of *P53* function. Previous studies have indicated that *P53* alterations might accelerate cancer development and could also lead to shorter survival in patients with *P53* mutated NSCLC [27]. Our findings first demonstrated the original *P53* mutation in *ALK* positive patients and this mutation of *P53* may correlate to the shorter PFS.

In summary, we conclude that*ALK* FISH and *EGFR*, *KRAS* mutational analysis on cytological specimens are sensitive methods for screening advanced stage NSCLC patients who are adequate for targeted treatment. Cytological specimens including FNA, FOB and PLE all provide feasible and effective material for the molecular analysis and further studies are required to validate the application in the routine molecular pathological diagnosis.

## MATERIALS AND METHODS

### Patients

One hundred and thirty-seven patients with advanced NSCLC were enrolled in this study from September 2013 to June 2015. All these cytological samples, including FNA, FPB and PLE, were tested for *ALK* fusion by FISH and 134 were performed with *EGFR* and *KRAS* mutational testing because 3 samples were not adequate for DNA extraction. Imaging data were independently reviewed by authors to evaluate their treatment responses according to the Response Evaluation Criteria in Solid Tumors (RECIST) version 1.1. PFS was calculated from the date of initiating TKI treatment to a radiologic or clinical observation of disease progression. The study protocol was approved by the Institute Review Board of the Cancer Hospital, Chinese Academy of Medical Sciences (CAMS), Beijing, China. The methods were carried out in accordance with the approved guidelines. Each participant signed an Institutional Review Board approved informed consent in accordance with current guidelines.

### Specimen preparation

The cytological specimen preparations were conducted according to a standard specimen processing protocol in our laboratory. The cases included FNA specimens obtained under image guidance with a cytopathologist present for adequacy assessment and pleural fluid specimens obtained by thoracentesis. The percentage of tumor cells more than 5% or over 500 tumor cells were used for DNA extraction and 100 tumor cells for ALK FISH analysis. The algorithm used for molecular testing in our study was depicted in Supplementary Figure S1. Each case had air-dried slides stained with Diff-Quik (DQ stain, Protocol Hema 3; Fisher Scientific, Kalamazoo, MI) and additional slides fixed in 95% alcohol for Papanicolaou staining. Fluid specimens also had a ThinPrep (Hologic, Marlborough, MA) slide prepared.

### Fluorescence *in situ* hybridization

FISH analysis was conducted as previously described [28]. Briefly, FISH analysis was performed using the Vysis LSI *ALK* Dual color, Break Apart Rearrangement Probe (Abbott/Vysis, Abbott Park, IL, USA). Samples were considered to be FISH positive if more than 15% of the scored tumour cells had split one or both *ALK* 5′ and 3′ probe signals or had isolated 3′ signals. Slides were evaluated independently by two experts blind to the patient's history and histological findings.

### *EGFR* and *KRAS* mutational testing

*EGFR* and *KRAS* mutational testing was conducted as previously described [29]. Briefly, mutational testing was performed using the Human*EGFR* or *KRAS* Mutation Qualitative Detection Kit (Beijing ACCB Biotech Ltd., China), which applies quantitative real-time PCR (qRT-PCR) platform combining amplification refractory mutation system (ARMS) primers and TaqMan probes. The sensitive mutations were defined as p.G719S/C/A, p.L858R, p.L861Q and insertions in exon 19. The hotspot mutations in *KRAS* gene were within codon 12 and 13 including p.G12C/V/S/R/D/A and p.G13D. The assay was carried out according to the manufacturer's protocol using the Stratagene Mx3000P real-time PCR system (Agilent technologies Inc, USA). Presence or absence of mutations was assessed from the fluorescence amplification curve.

### Targeted next-generation sequencing

For one patient who did not response well to crizotinib treatment, targeted next-generation sequencing was performed. Genomic DNA was profiled by using a capture-based targeted sequencing panel (Burning Rock Biotech, Guangzhou, People's Republic of China). In brief, human genomic regions of 271 kb, including all exons in 56 genes and selected introns in *ALK*, *RET* and *ROS1* for the detection of translocation events, were captured by using 120-bp probes and were sequenced (Supplementary Table S4). The concentration of the DNA samples was measured with the Qubit dsDNA assay. Fragments of 200 to 400-bp sizes were selected with beads (Agencourt AMPure XP kit; Beckman-Coulter, Brea, CA), followed by hybridization with the capture probes baits, hybrid selection with magnetic beads, and PCR amplification. A bioanalyzer high-sensitivity DNA assay was then used to assess the quality and size range. Available indexed samples were then sequenced on a Nextseq (Illumina, San Diego, CA) with pair-end reads. Sequence data were analyzed by GATK 3.2 (https://www.broadinstitute.org/gatk/) and DNA translocation analysis was performed by using both Tophat2 (https://ccb.jhu.edu/software/tophat/index.shtml) and Factera 1.4.3 ((http://factera.stanford.edu) [30].

### Statistical analysis

Differences of patient characteristics and clinicopathologic factors in the two-dimensional cross-comparison were evaluated statistically by Pearson's χ^2^-test orFischer's exact test. Statistical tests were two-sided, and *P* < 0.05 was considered significant. Estimation of PFS was calculated using the Kaplan–Meier method. Statistics were carried out using SPSS software (version 16.0 of SPSS, Chicago, IL, USA).

## SUPPLEMENTARY FIGURE AND TABLES


