# Supplementary material for: Assessment of cytology based molecular analysis to guide targeted therapy in advanced non-small-cell lung cancer
Source: Oncotarget. 2015 Dec 18;7(7):8332–40. doi: 10.18632/oncotarget.6671 (PMC4884996; doi:10.18632/oncotarget.6671)
Supplement: Supplementary file 1 [file oncotarget-07-8332-s001.pdf]

SUPPLEMENTARY FIGURE AND TABLES

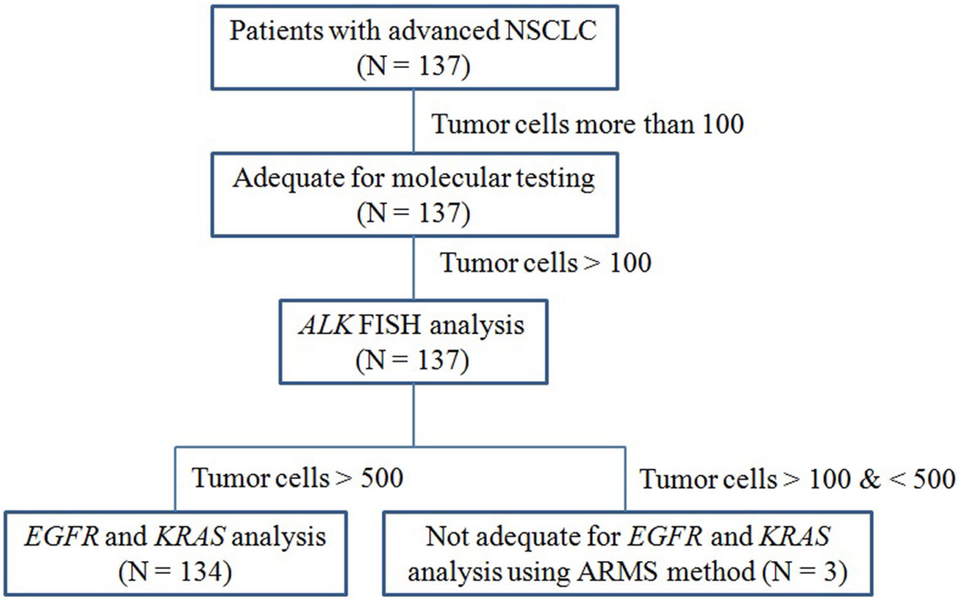

Supplementary Figure S1: The algorithm used for molecular testing in the study.

Supplementary Table S1: Correlation between the *ALK* FISH analysis results in cytological samples

Type of samples	Total (n = 137)	Positive (n = 16)	Negative (n = 121)	*P* value
FNA	91 (66.4%)	12 (75.0%)	79 (65.3%)	0.32
FOB	5 (3.6%)	1 (6.2%)	4 (3.3%)	
PLE	41 (30.0%)	3 (18.8%)	38 (31.4%)	

Abbreviations: *ALK* = anaplastic lymphoma kinase; FISH = fluorescence in situ hybridization; FNA = fine needle aspiration; FOB = fibreoptic bronchoscopic; PLE = pleural effusion.
P value: Fisher's exact test

Supplementary Table S2: Correlation between the *EGFR and KRAS* mutational analysis results in cytological samples

Type of samples	*EGFR*		*P* value	*KRAS*		*P* value
	Total (n = 134)	Positive (n = 60)		Total (n = 134)	Positive (n = 8)	
FNA	90 (67.2%)	36 (60.0%)	0.41	90 (67.2%)	6 (75.5%)	1.0
FOB	5 (3.7%)	1 (1.7%)		5 (3.7%)	0	
PLE	39 (29.1%)	23 (38.3%)		39 (29.1%)	2 (25.0%)	

Abbreviations: FNA = fine needle aspiration; FOB = fibreoptic bronchoscopic; PLE = pleural effusion.
P value: Fisher's exact test

Supplementary Table S3: Clinicopathologic characteristics of *EGFR* and *KRAS* mutational cytology cases

Characteristics	*EGFR* positive (n = 60)	*EGFR* negative (n = 74)	*P* value	*KRAS* positive (n = 8)	*KRAS* negative (n = 126)	*P* value
Sex			0.002†			0.26§
Male	15 (28.3%)	38 (71.7%)		5 (62.5%)	48 (38.1%)	
Female	45 (55.6%)	36 (44.4%)		3 (37.5%)	78 (61.9%)	
Histologic type			NA			NA
ADC	57 (95.0%)	66 (89.2%)		5 (55.6%)	118 (93.6%)	
SCC	2 (3.3%)	1 (1.4%)		0	3 (2.4%)	
Adenosquamous	0	1 (1.4%)		0	1 (0.8%)	
NSCLC, NOS	1 (1.7%)	6 (8.1%)		4 (44.4%)	4 (3.2%)	
Age			0.40‡			0.67‡
Mean (SD)	59.7 ± 10.6	57.9 ± 13.3		57.0 ± 12.3	58.8 ± 12.2	
Median	61.0	57.5		57.5	59.0	
Range	37.0 - 81.0	27.0 - 85.0		31.0 - 71.0	27.0 - 85.0	

Abbreviations: ADC = adenocarcinoma; SCC = squamous cell carcinoma; NSCLC, NOS = non-small cell lung cancer, not otherwise specified.
†Two-sided χ^2 test
‡Two-sided Kruskal Wallis test
§Fischer's exact test

Supplementary Table S4: Fifty-six genes in targeted next-generation sequencing panel

Gene No.	Targeted DNA	Gene No.	Targeted DNA
1	AKT1	29	HRAS
2	ALK	30	JAK1
3	ARAF	31	JAK2
4	ATM	32	KDR
5	BIM	33	KIT
6	BRAF	34	KRAS
7	BRCA1	35	MAP2K1
8	BRCA2	36	MET
9	CCND1	37	MTOR
10	CDK4	38	NF1
11	CDK6	39	NRAS
12	CDKN2A	40	NRG1
13	CYP2C19	41	NTRK1
14	CYP2D6	42	NTRK2
15	CYP3A4	43	NTRK3
16	DDR2	44	PDGFRA
17	DPYD	45	PIK3CA
18	EGFR	46	PTCH1
19	ERBB2	47	PTEN
20	ERBB3	48	RAF1
21	ERBB4	49	RET
22	FGF3	50	ROS1
23	FGF4	51	SMO
24	FGF19	52	STK11
25	FGFR1	53	TP53
26	FGFR2	54	TSC1
27	FGFR3	55	TSC2
28	FLT3	56	UGT1A1
